# Supplementary material for: Factors influencing perceived importance of haemodialysis adherence among end stage kidney failure patients in tertiary care centres in Nepal
Source: PLoS One. 2026 Jun 17;21(6):e0351702. doi: 10.1371/journal.pone.0351702 (PMC13274901; doi:10.1371/journal.pone.0351702)
Supplement: Attachment — Submitted filename: Response to Reviewer_PONE-D-26-07737.pdf [file pone.0351702.s003.pdf]

PONE-D-26-07737

Factors influencing perceived importance of hemodialysis adherence among end stage renal disease patients in tertiary care centers in Nepal

PLOS One

May 3, 2026

The Academic Editor,
Plos One

Re: Factors influencing perceived importance of hemodialysis adherence among chronic kidney failure patients in tertiary care centers in Nepal

Dear Dr. Rahman,

Thank you for considering publishing our manuscript. We also thank you and the reviewers for your constructive comments, as they have tremendously improved the quality of the manuscript. We have taken note of all the reviewers' comments and made corrections accordingly.

Please find below a point-by-point response to each comment raised.

Thank you for your time and consideration.

Sincerely,

Pramita Shrestha

<u>Response to the Line-by-Line review below</u>
Title
The title is acceptable but could be simplified.
Suggested alternative:
"Factors associated with perceived importance of haemodialysis adherence among patients with end-stage kidney disease (or chronic kidney failure) in Nepal."
Response: As suggested we changed the title to "Factors associated with perceived importance of haemodialysis adherence among patients with chronic kidney failure in Nepal."

Abstract
Lines 18–22
The wording is unclear and contains multiple grammatical errors.
Example:
"Perception towards importance of adhering to hemodialysis affects treatment adherence."
This statement requires citation and clarification.
Response: Line number 19-22. It has been restated as: Perception of the importance of adhering to dialysis helps to determine how patients interpret and interact with the influencing attitude, thoughts and behaviors towards adhering to dialysis.

Introduction
Lines 39–47
The introduction contains repetition and grammatical errors, including duplicated phrases.
Example:
"low-to middle-income patients' needs renal replacement therapy…"
This sentence should be rewritten for clarity.
Response: Line number 41-47. The sentence was rewritten as:
Chronic Kidney Disease (CKD) is a significant global public health concern that can lead to chronic kidney failure, requiring costly treatments such as dialysis or renal transplantation for survival. Chronic renal failure contributes to burden in health care system, productivity and economic growth (1). Worldwide, an estimated 4.9 to 9.7 million chronic renal failure patients need kidney replacement therapy, with majority residing in low-to-middle income countries. (2,3). In Nepal, CKD accounted for approximately two per cent of total deaths and one per cent of total DALYs, placing a significant financial burden on patients, the health care system and society.

Line 48–49
"Renal transplantation relies highly on the intense availability…"
This statement is unclear and requires revision.
Response : At line number 48-51. The statement has been revised to: Renal transplantation

requires substantial resources such as donor availability and long-term financial support. Therefore, dialysis is used as primary treatment option for patients with chronic renal failure.

General comment
The introduction would benefit from:
• A clearer knowledge gap
• Explanation of why perception matters
Response : Line number 63-72. Despite growing evidence on treatment adherence among hemodialysis patients, most studies have focused on clinical and socio-demographic factors, with limited exploration of patients' perceptions regarding the importance of adherence, particularly in low- and middle-income countries like Nepal. Patients' perceptions play a important role in shaping their adherence behavior, as they influence attitudes, motivation, and engagement with treatment. Understanding these perceptions is essential for developing targeted interventions to improve adherence outcomes. Therefore, this study aims to assess the factors influencing the perceived importance of adherence to hemodialysis among patients with chronic renal failure. The findings contribute evidence on patient's perception regarding the importance of dialysis and may inform policies and health systems to plan and implement effective interventions that improve adherence.

Methods
Lines 68–70
"perception on of the importance of adherence"
This phrase appears multiple times and should be corrected to:
"perception of the importance of adherence."
Response : The phrase was corrected to: "perception of the importance of adherence.".

Study sites
The authors should explain why these two centers were selected and whether they are representative of dialysis services in Nepal.
Response: At line number 86-88: These tertiary hospitals were selected because they provide specialized care for patients with chronic renal failure undergoing hemodialysis and have patient flow from diverse geographical and socioeconomic backgrounds that enhance representativeness.

Questionnaire
More detailed information is needed regarding the following aspects:
- Translation procedures
- Interviewer training
- Questionnaire administration
- Validation steps
Response:

Line number 150-153: Translation procedures: The standard End stage Renal Disease Adherence Questionnaire was available in English language and translated into Nepali to ensure linguistic appropriateness. Translation was done by expert. The translated Nepali version was then retranslated into English by another translator not involved in initial translation. Differences observed were reviewed and solved by research team.
Line number 155-158: Interviewer training: The training for data collection was provided to the enumerators before starting data collection. Training was given on study objectives, inclusion and exclusion criteria of the patients, informed consent process, confidentiality and interview techniques to ensure consistency and minimize interviewer bias.
Questionnaire administration: Participants were selected based on predefined inclusion criteria. The questionnaire was administered in Nepali language. Data was collected using face to face interview that lasted for about 30- 35 minutes. Verbal and written consent was obtained from each of the participants prior to data collection and they were informed about the study objectives. Participants were informed about their rights to participate in study or not. Participant privacy was maintained throughout the study.
Validation steps:
Line number 160-163: Pretesting of the quantitative questionnaire was conducted among 17 hemodialysis patients of Methinkot Hospital, Kavrepalanchok, and patients from the surgery outpatient department of DHKUH visited for their fistula treatment. After pretesting, the tools were modified and the questions were restructured to simplify and make the participants understand and use them correctly. Content validity was ensured through expert review by nephrologists.

Statistical Methods
The description of statistical analysis is insufficient. Please include the following information:
- Type of regression model used
- Criteria for variable inclusion
- Approach to handling missing data

Response: Line number 169-182. Poisson regression was used to assess the association between perception on the importance of adherence to dialysis and sociodemographic, dialysis-related factors and accessibility related factors, adjusting for sex, residence, marital status, religion, educational status, occupation and family source of income. Prior to multivariate analysis, multicollinearity was assessed using the variation inflation factor. High multicollinearity with VIF>10 was seen in the variable frequency of dialysis sessions per week. Pairwise correlation was examined but did not show correlation of variable frequency of dialysis session per week with other exposure variables and therefore predicted this was due to combination of other independent variables. Therefore, frequency of dialysis session per week was excluded in final multivariate model .Crude and adjusted prevalence ratios were reported within 95% confidence

interval, and statistical significance was decided if the p-value was <0.05. Data completeness was checked at the time of collection to minimize missing information so we did not have any missing data.

Results

The results section is generally understandable but requires improvement in the following areas:

- Clearer presentation of tables

- Consistent reporting of confidence intervals

- A more organized narrative structure

Response: The result section has been streamlined.

Discussion

The discussion section should be significantly expanded. Important components that are currently missing include:

- A comparison with international studies on dialysis adherence

- Interpretation in the context of health system limitations in Nepal

- Acknowledgment of the study's limitations

Response: Line number 255-263: This study found no significant association between perception of the importance of adherence to dialysis and factors including gender, residence, religion, education, current dialysis access site, comorbid condition, complications during dialysis, number of medications taken as prescribed, and intradialytic weight gain. A systematic review revealed that illness perception was associated with domains of adherence especially to diet and fluid restriction but not to dialysis sessions (30). A study from Palestine and Rawanda revealed age, gender and residence were positively associated with dialysis adherence score (15, 31). Even though, the Nepal Government has been offering free dialysis services since 2016, not all hemodialysis patients consistently follow their treatment plans. More than one fourth of the people have faced catastrophic health expenditure for dialysis (27,28).Studies suggest that a large proportion of chronic renal failure patients struggle with adherence to treatment regimen due to financial constraints, lack of awareness, and limited access to treatment facilities (29).The lack of association between these variables in the study could be due to a relatively small sample size and other possible residual confounders. We had done acknowledgement of study limitations 258-265.

Limitations

The following limitations should be explicitly stated:

1. Cross-sectional design

2. Bias from self-reported questionnaires

3. Limited generalizability beyond tertiary centers

4. Measurement of perception and practice, rather than actual adherence

Response: The given limitations were stated in line number 274-286. The study was conducted in only two tertiary care centres in Nepal, which may not have adequately represented all the hospitals throughout Nepal, which could affect the generalizability of results. Secondly, the cross-sectional design might have been limited in generating causal inference, as we were not able to know if the perception towards dialysis adherence was already improved, or it was improved only after getting exposed to the dialysis treatment-related exposures. The majority of the questions were self-reported by the participants, establishing social desirability or recall bias as a fourth limitation of the study. Also, the study might have residual confounders that might have distorted the effect measures. Moreover, the study primarily measured perception of patients on adherence and self-reported practices related to adherence, which may not completely reflect their actual treatment adherence behavior. This can be applied to qualitative studies; future studies may consider doing so for in-depth information on perceptions.
