# Supplementary material for: Factors influencing perceived importance of haemodialysis adherence among end stage kidney failure patients in tertiary care centres in Nepal
Source: PLoS One. 2026 Jun 17;21(6):e0351702. doi: 10.1371/journal.pone.0351702 (PMC13274901; doi:10.1371/journal.pone.0351702)
Supplement: Attachment — Submitted filename: Perceived importance of hemodialysis adherence .doc [file pone.0351702.s001.doc]

**Factors influencing perceived importance of haemodialysis adherence among end stage renal disease patients in tertiary care centres in Nepal**

Pramita Shrestha^1,2*#^, Anisha Koirala^2*^, Prajwal Satyal^3^, Ashish Mani Bhurtel^4^, Biraj Man Karmacharya^2^

^1^Department of Epidemiology, School of Public Health, University of Washington, USA

^2^Department of Public Health, Kathmandu University School of Medical Sciences, Nepal

^3^Department of Agriculture Economics, University of Hohenheim, Stuttgart, Germany

^4^Department of Internal Medicine, Silverline Hospital, Kathmandu, Nepal

^*^ Joint first authors

^#^Corresponding author

[pramitasth@kusms.edu.np](mailto:pramitasth@kusms.edu.np)

**ABSTRACT**

**Background:** Chronic Kidney Disease is a significant global public health concern requiring renal replacement therapy such as dialysis. Perception towards importance of adhering to hemodialysis affects treatment adherence. This study aimed to assess the perception of the importance of adherence to hemodialysis and its associated factors among end-stage renal disease patients in tertiary care centres in Nepal.

**Methods:** This was a cross-sectional study conducted from May to August 2025 in two tertiary care centres in Nepal that included patients undergoing hemodialysis aged 18 years and above. The outcome variable was perception of the importance of adherence to dialysis and was measured using The End Stage Renal Disease Adherence Questionnaire (ESRD-AQ). The independent variables were socio-demographics, dialysis-related characteristics and accessibility factors.

**Results:** Among 283 hemodialysis patients in the study, their mean age was 50.6 years with higher prevalence of good perception (91%) among single [APR (adjusted prevalence ratio):1.12; 95% CI (confidence interval): 1.03-1.21)] and parents accompanying patients (APR:1.14; 95% CI:1.01-1.28) compared to married, and non-parent accompanying patients respectively.

**Conclusion:** The study showed good perception of hemodialysis adherence, especially among singles and those accompanied by their parents.

**Keywords:** *adherence, end-stage renal disease, hemodialysis, perception, prevalence ratio*

**INTRODUCTION**

Chronic Kidney Disease (CKD) is a significant global public health concern, leading to end stage renal disease (ESRD), requiring costly treatments like dialysis or renal transplantation for survival. It also significantly affects health care expenses, productivity and growth (1). Worldwide, an estimated 4.9 to 9.7 million ESRD patients need kidney replacement therapy, with the majority residing in low-to middle-income countries (2,3). In Nepal, CKD accounted for approximately two per cent of total deaths and one per cent of total DALYs, placing a significant financial burden on patients, the health care system and society (4,5). Renal transplantation relies highly on the intense availability and affordability of adequate resources. Hence, dialysis is commonly preferred till time. (6,7) The effectiveness of treatment depends on adherence to treatment regimen as prescribed by the physician.(8,9)

More than half of the patients undergoing dialysis from a study in Northern Indian showed good treatment adherence, which was influenced by patients’ perceptions of treatment and counselling frequency.(10) Perception of the patients on the importance of adhering to the CKD treatment affects the treatment adherence in hemodialysis patients.(10,11) Non adherence to treatment regimen increases risks of morbidity and mortality (12,13). Perception of the importance of adhering to dialysis helps to determine how patients interpret and interact with the influencing attitude, thoughts and behaviours towards adhering to dialysis (14,15). Good perception encourages patients to receive effective dialysis treatment (16). Perception of dialysis adherence can be modified with interventions such as awareness sessions, counselling, peer support and revisiting health systems (14,15,). Due to the limited number of studies in this area, this study aimed to assess the factors influencing the perceived importance of adherence to hemodialysis among end-stage renal disease patients in two tertiary care centres in Nepal. The findings contribute evidence on patient’s perception regarding the importance of dialysis and may inform policies and health systems to plan and implement effective interventions that improve adherence.

**METHODS**

**Study design**

This was a cross-sectional hospital-based study conducted to assess the perception of the importance of adherence to dialysis and its associated factors among end-stage renal disease patients from two tertiary care centres in Nepal. The study was conducted from May to August 2025.

**Study site and justification**

The study was conducted at two different tertiary care centres in Nepal: Dhulikhel Hospital-Kathmandu University Hospital (DHKUH), located in Dhulikhel, Kavrepalanchowk district and Sahid Dharmabhakta National Transplant Centre (SDNTC) situated in Bhaktapur district of Bagmati province. Each patient had a different schedule of dialysis sessions: once, twice, or three times a week.

**Study population**

The study included patients with end-stage renal disease who had been receiving hemodialysis at DHKUH and SDNTC for at least 3 months. The study selected all eligible patients attending dialysis sessions on the day of data collection ~~and~~ who provided written informed consent to participate. The study selected adults aged 18 years and above, diagnosed with stage five CKD, undergoing hemodialysis and willing to participate voluntarily in the study. Moreover, the participants were excluded if the patients were critically ill or were under peritoneal dialysis.

**Sampling technique**

In each hospital, the list of patients receiving hemodialysis was obtained from their medical records. The study approached patients attending dialysis sessions on the day of data collection. Hemodialysis patients were selected consecutively at DHKUH and SDNTC until the planned sample size was reached.

**Sample Size**

The population parameter in the study was a proportion, so the sample size was calculated using the standard formula for a single proportion: n = z²pq/d².(17) Assuming 63% hemodialysis adherence from a study conducted in Nepal (8), at a 95% level of significance and 5.6% error, the total calculated sample size was 283. Out of these, 50 participants belonged to DHKUH, and 233 participants were included from SDNTC. The following is the computation of how this number was obtained.

**Study variables**

*Dependent variables*

The study included “perception on the importance of adherence to dialysis” as the dependent variable and was measured by the End Stage Renal Disease Adherence Questionnaire (ESRD-AQ), which consisted of 46 questions divided into five sections (18). The fourth part of the questionnaire included questions on information for perception on each treatment regimen. The answers to the perception questions about the dialysis schedule were categorised on a 5-point Likert scale: very important, important, moderately important (3), slightly important, and not important. Perception of the importance of adherence to dialysis was then recategorised into good perception (responses of very important and important; scores 5 and 4) and *poor perception* (responses of moderately important, little important, and not important; scores 3-1).

***Independent variables***

The independent variables used in the study were grouped into three domains: sociodemographic variables, dialysis-related characteristics and accessibility factors.

Socio-demographic characteristics

This included age (in completed years), sex~~gender~~, place of residence (urban/rural), religion, education status, family sources of income and marital status. Agriculture referred to subsistence or commercial farming and livestock rearing, which is distinct from service (formal salaried employment in government or private institutions) and daily wage labor (informal, short-term, wage-based work without a fixed salary). Business was retained as a separate category for self-employed, income-generating activities. Overlapping and ambiguous categories were merged where appropriate to improve interpretability.

Dialysis-related characteristics

This comprised the length of dialysis treatment (hours), frequency of dialysis sessions per week and presence of comorbidities.

Accessibility factors

The accessibility factors in the study included mode of transportation (private transport, bus, taxi, ambulance, others), distance from the dialysis center (in kilometers), accompany to dialysis center (myself, parents, spouse, child, friend, others), hemodialysis access history was defined as any previous vascular access used prior to the current access, while current dialysis access site was categorized as arteriovenous fistula, temporary catheter, or permanent catheter).

**Data collection tools**

The study collected quantitative data employing face-to-face interviews utilising a structured questionnaire. The End Stage Renal Disease Adherence Questionnaire (18) was employed to determine perception and adherence levels, and a structured survey from NCD Steps Survey 2019 (19) and Nepal Living Standard Survey (20) was utilised to gather socio-demographic characteristics and medical and non-medical expenditure of the patients, respectively. Furthermore, a structured questionnaire was prepared to capture accessibility factors and household characteristics. The study also reviewed clinical records to capture dialysis-related characteristics.

**Reliability and validity of the tool**

The standard tools were used after obtaining the authors' permission. The questionnaire was translated into Nepali and back-translated into English to ensure reliability. The questionnaires were also reviewed by a Nephrologist from DHKUH and filled out by the research team and trained research assistants. Pretesting of the quantitative questionnaire was conducted among 17 hemodialysis patients of Methinkot Hospital, Kavrepalanchok, and patients from the surgery outpatient department of DHKUH visited for their fistula treatment. The study modified the pre-tested questions as required.

**Data management and analysis**

The study collected data using Kobo Toolbox and then exported it to STATA 14.2 for statistical analysis and further cleaning. The perception on importance of adherence to dialysis was presented into frequency and percentage. The sociodemographic factors, dialysis-related factors and accessibility related factors were presented in frequency and percentage for categorical variables and mean and standard deviation for numeric variables. Poisson regression was used to assess the association between perception of the importance of adherence to dialysis and sociodemographic, dialysis-related factors and accessibility related factors,, adjusting for sex~~gender~~, residence, marital status, religion, educational status, occupation and family source of income. Prior to multivariate analysis, multicollinearity was assessed using the variation inflation factor. High multicollinearity with VIF>10 was seen in the variable frequency of dialysis sessions per week. Crude and adjusted prevalence ratios were reported within95% confidence interval, and statistical significance was decided if the *p*-value was <0.05.

**Bias**

The study might have introduced selection bias, as it was not possible to adapt probability sampling. The self-reported information might have generated social desirability bias and/or recall bias in answering the questions that they were asked. Also, the study could not include all the possible confounders, resulting in inclusion of residual bias leading to underestimated or overestimated effect measures.

**Ethical approval**

The protocol was approved by the Kathmandu University Institutional Review Committee (KU-IRC) on 6 April 2025, with approval number 72/25. Permissions were taken from Shahid Dharma Bhakta National Transplant Center and Dhulikhel Hospital. Verbal and written consent was obtained from each of the participants prior to data collection, and they were informed about their rights to participate in the study or not. Participant privacy and confidentiality were maintained throughout the study, ensuring their voluntary participation.

**RESULTS**

**Sociodemographic and dialysis related characteristics**

Tables 1 and 2 summarise the sociodemographic and dialysis-related characteristics of the participants, respectively. A total of 283 hemodialysis patients were included with a mean age of 50.6 ± 14.6 years. Most of the participants were male, married, mostly residing in urban settings, belonged to the Hindu religion, attended secondary or higher education, and their main source of family income was business. The majority did not have any companions to the dialysis centre, lived within 5 kilometres, and used private transportation to reach it.

Most of the participants received dialysis for the standard duration of 4 hours, were on dialysis sessions twice a week and used AV fistula for dialysis currently, while having both arteriovenous fistula and catheter as hemodialysis access history. Around two-fifths of the participants had the presence of one comorbidity, and the majority of them adhered to dialysis and had a good perception of its importance.

**Factors associated with the perception on importance of adherence to dialysis schedule**

In the study, about 91% of the patients under hemodialysis had a good perception of the importance of adherence to the dialysis schedule. Table 3 summarises the association of sociodemographic factors, dialysis-related characteristics and access-related characteristics with perception of the importance of adherence to the dialysis schedule. In the multivariate model, the participant with single status had 9 % higher prevalence of good perception towards the importance of adherence to dialysis compared to the married (APR:1.09; 95% CI: 1.01-1.21; *P* =0.005). Participants who reached the dialysis centre with their parents had 14 % higher prevalence of good perception compared to those accompanied by others (APR: 1.14; 95% CI: 1.01–1.28; p = 0.02).

**Table 1: Socio-demographic characteristics of the patients under dialysis (n=283)**

| **Characteristics** | **Frequency** |
| --- | --- |
|  | **n (%)** |
| **Age in years** (Mean ± SD) | 50.57 ± 14.63 |
| **Gender** |  |
| Male | 185 (65.4) |
| Female | 98 (34.6) |
| **Residence** |  |
| Urban | 223 (78.8) |
| Rural | 60 (21.2) |
| **Marital Status** |  |
| Married | 231 (81.6) |
| Single | 52 (18.4) |
| **Religion** |  |
| Hindu | 231 (81.6) |
| Others (Buddhist, Muslim, Christian) | 52 (18.4) |
| **Educational Status** |  |
| No formal schooling | 64 (22.6) |
| Less than primary and primary | 91 (32.2) |
| Secondary and above | 128 (45.2) |
| **Family Source of Income* (NRs)** |  |
| Agriculture | 68 (24.0) |
| Business | 94 (33.2) |
| Daily Wage | 43 (15.2) |
| Foreign Employment | 46 (16.2) |
| Service | 65 (22.9) |
| **Accompany to dialysis center*** |  |
| None | 135 (47.7) |
| Spouse | 103 (36.4) |
| Others (child/brother/sister/daughter in law) | 77 (27.2) |
| Parents | 13 (4.6) |
| **Distance to reach to dialysis center** |  |
| <5 km | 141 (49.8) |
| 5-20 km | 126 (44.5) |
| 21-50 km | 16 (5.6) |
| **Mode of transport** |  |
| Private transport (private/taxi) | 148 (52.3) |
| Public transport | 84 (29.7) |
| Walking | 51 (18) |

*Multiple response; SD: Standard deviation, IQR: Interquartile range; NRs: Nepali currency; km: Kilometers

**Table 2 Dialysis-related characteristics of the patients under dialysis** (n=283)

| **Characteristics** | **Frequency** |
| --- | --- |
|  | **n (%)** |
| **Length of dialysis treatment** |  |
| 4 hours | 267 (94.4) |
| Less than 4 hours | 16 (5.6) |
| **Frequency of dialysis in a week** |  |
| Twice a week | 181 (63.96) |
| Thrice a week | 95 (33.57) |
| Once a week | 7 (2.47) |
| **Comorbid condition** |  |
| No comorbidity | 34 (12.0) |
| Presence of one comorbidity | 116 (41.0) |
| Presence of two comorbidity | 89 (31.5) |
| Presence of three or more comorbidity | 44 (15.5) |
| **Perception on following dialysis schedule** |  |
| Good perception | 258 (91.2) |
| Poor perception | 25 (8.8) |
| **Perception on importance of dialysis adherence** |  |
| Extremely important | 218 (77.1) |
| Very important | 40 (14.1) |
| Moderately important | 12 (4.2) |
| Slight important | 7 (2.5) |
| Not important | 6 (2.1) |
| **HD access history** |  |
| Only AVF | 81 (29.0) |
| AVF + any catheter  Only catheter | 198 (71.0)  4 (1.41) |
| **Current HD access site** |  |
| AV fistula | 266 (94.0) |
| Temporary Catheter | 14 (4.2) |
| Permanent Catheter | 6 (1.8) |

IQR: Interquartile range; *Multiple response; km = kilometer; HD: hemodialysis; AVF: arteriovenous fistula; AV: Arteriovenous: Good perception was defined as respondents rating the importance of adherence to dialysis as “very important” or “important” (Likert scale scores 5 or 4) on the ESRD Adherence Questionnaire (ESRD-AQ).

**Table 3: Factors associated with perception on importance of adherence to dialysis of the patients under hemodialysis (n=283)**

| **Variables** | **cPR (95% CI)** | **p-value** | **aPR (95%CI)** | **p-value** |
| --- | --- | --- | --- | --- |
| **Gender** |  |  |  |  |
| Male | Ref |  |  |  |
| Female | 1.06 (0.88-1.18) | 0.07 | 1.04 (0.98-1.10) | 0.12 |
| **Residence** |  |  |  |  |
| Urban | Ref |  |  |  |
| Rural | 1.03 (0.95- 1.12) | 0.46 | 1.02 (0.93-1.13) | 0.58 |
| **Marital Status** |  |  |  |  |
| Married | Ref |  |  |  |
| Single | 1.09 (1.03- 1.16) | 0.002 | **1.12 (1.03-1.21)** | **0.005** |
| **Religion** |  |  |  |  |
| Hindu | Ref |  |  |  |
| Others (Buddhist, Muslim, Christian) | 1.01 (0.93 – 1.11) | 0.74 | 0.99 (0.89-1.09) | 0.88 |
| **Educational Status** |  |  |  |  |
| No formal schooling | Ref |  |  |  |
| Less than primary and primary | 0.95 (0.86- 1.06) | 0.37 | 0.96 (0.87-1.06) | 0.51 |
| Secondary and above | 1.01 (0.92-1.09) | 0.85 | 0.99 (0.89-1.10) | 0.93 |
| **Family source of income***** |  |  |  |  |
| Agriculture | 1.04 (0.97- 1.12) | 0.27 | 0.92 (0.79-1.06) | 0.28 |
| Business | 0.94 (0.86- 1.02) | 0.16 | 0.86 (0.70-1.02) | 0.08 |
| Daily wage | 0.93 (0.82- 1.06) | 0.29 | 0.84 (0.70-1.01) | 0.07 |
| Foreign employment | 0.97 (0.87- 1.08) | 0.59 | 0.86 (0.72-1.03) | 0.10 |
| Service | 1.03 (0.96- 1.12) | 0.33 | 0.92 (0.78-1.08) | 0.35 |
| **HD access history** |  |  |  |  |
| Only AVF | Ref |  |  |  |
| AVF+ any catheter | 0.96 (0.89- 1.03) | 0.25 | 1.03 (0.94-1.12) | 0.51 |
| Only catheter | 1.07 (1.00-1.12) | 0.02 | 1.12 (0.93-1.36) | 0.20 |
| **Mode of Transport** |  |  |  |  |
| Private transport (Private/Taxi) | Ref |  |  |  |
| Public transport (Bus) | 0.99 (0.90 -1.08) | 0.89 | 0.97 (0.88-1.06) | 0.53 |
| Walking | 1.09 (1.02-1.16) | 0.01 | 1.05 (0.94-1.18) | 0.32 |
| **Accompany to dialysis center†** |  |  |  |  |
| None † | 1.08 (1.00- 1.15) | 0.04 | 1.07 (0.97-1.17) | 0.13 |
| Parents † | 1.10 (1.06- 1.14) | <0.001 | **1.14 (1.01-1.28)** | **0.02** |
| Spouse † | 1.00(0.93- 1.08) | 0.96 | 1.06 (0.96-1.17) | 0.18 |
| Others † | 0.92 (0.83- 1.01) | 0.09 | 0.98 (0.88-1.09) | 0.72 |

PR: Prevalence ratio; cPR: crude prevalence ratio; aPR: adjusted prevalence ratio; CI: Confidence Interval; HD: hemodialysis; AVF: arteriovenous fistula; NRs: Nepali currency; Adjusted for gender, residence, marital status, religion, education, occupation, family source of income; ***This is a variable with multiple response; hence reference is “no” for each option of family source of income; **†:** the reference group for these variables are no for each options (none, parents, spouse, others).

**DISCUSSION**

The findings showed that the majority of the participants (91.17%) had a good perception of the importance of adherence to dialysis. This finding was consistent with the study conducted in North India, Pakistan, Palestine and Yemen, showing good perception of following dialysis schedule (10, 15,21, 22). The patients under dialysis are in continuous interaction with the health personnel, receiving counselling and instructions from them, enhancing positive perception.(23)

This study showed that participants who were single and accompanied by parents to the dialysis centre had a positive perception, and the findings were consistent with other studies from Malaysia and other settings.(24,25,26) In the Nepalese context, although marital status and parents’ accompaniment are related, they correspond to distinct dimensions of social support. Being married does not necessarily imply active involvement of parents in dialysis care, whereas accompaniment during dialysis sessions reflects functional support. Hence, higher adherence among single participants of the study might have occurred due to more self-management practices and independence. Moreover, parental accompaniment might have improved adherence through their emotional and logistical support.

This study found no significant association between perception of the importance of adherence to dialysis and factors including gender, residence, religion, education, current dialysis access site, comorbid condition, complications during dialysis, number of medications taken as prescribed, and intradialytic weight gain. The lack of association between these variables in the study could be due to a relatively small sample size and other possible residual confounders.

The study team did not provide any additional counselling or explicit warnings regarding the consequences of missing hemodialysis sessions prior to or during the data collection period. Due to the observational, cross-sectional nature of the study, the perception was based on the regular care they have been receiving from their health professionals. However, the absence of structured counselling about the probable outcomes of missed dialysis sessions may have prompted patients’ adherence and perception. Future studies should explore whether targeted patient education and counselling interventions can improve adherence to haemodialysis schedules.

The study was conducted under a few limitations. Firstly, the study was conducted in only two tertiary care centres in Nepal, which may not have adequately represented all the hospitals throughout Nepal, which could affect the generalizability of results. Secondly, the cross-sectional design might have been limited in generating causal inference, as we were not able to know if the perception towards dialysis adherence was already improved, or it was improved only after getting exposed to the dialysis treatment-related exposures. The majority of the questions were self-reported by the participants, establishing social desirability or recall bias as a fourth limitation of the study. Also, the study might have residual confounders that might have distorted the effect measures. Moreover, the study may not have measured perception completely as a quantitative variable. This can be applied to qualitative studies; future studies may consider doing so for in-depth information on perceptions. The categorization of the perception score may have introduced loss of data granularity reducing variability and statistical power. Future studies may use alternative ordinal or continuous modelling methods for more nuanced insights. Some variables were collected as multiple-response items, which might have limited the ability to model them as mutually exclusive categories. The use of convenient consecutive sampling might have introduced selection bias, limiting the generalizability of the findings. Despite limitations, the study had strengths as well. The study was the first of its kind in Nepal, conducted at two tertiary care centres, where patients undergoing dialysis were interviewed to learn about their perceptions of the significance of dialysis. This will provide insightful evidence and inform programs or studies to focus on enhancing their perceptions as well as to minimise the psychosocial burden of the patients who are already under stress to attend continuous dialysis sessions.

**Conclusion**

Patients under hemodialysis or hemodialysis had a good perception of the importance of adherence to dialysis, particularly for those who were still single and those accompanied by their parents. The findings highlighted that personal independence and social support play a significant role in adherence to the dialysis schedule. The study recommends incorporating interventions such as counselling, peer support, awareness sessions and health education that can enhance the positive perception of the significance of adherence to dialysis, leading to increased adherence to dialysis, which can help prolong the patient’s life. Also, future research should encompass the qualitative perspective on the perception of the importance of adherence to the dialysis schedule to gain deeper insights.

**Conflict of Interest**

The authors declare no conflicts of interest.

**Author Contributions**

Conceptualization: PS, AK, and BMK; methodology: PS, AK, PST, AB BMK; investigation: PS, AK, PST, AB BMK; data analysis: PS, AK, PST; writing—original draft preparation: PS, AK; writing—review and editing, PS, AK, PST, AB BMK; supervision: BMK.

**Funding**

This study did not receive any funding.

**Acknowledgements**

We would like to acknowledge the support from the teams DHKUH and SDNTC and all the participants in the study.

**REFERENCES**

1. Fiseha T, Osborne NJ. Burden of end-stage renal disease of undetermined etiology in Africa. Ren Replace Ther. 2023 Sept 5;9(1):44. DOI:10.1186/s41100-023-00497-w. URL: https://doi.org/10.1186/s41100-023-00497-w

2. Liyanage T, Ninomiya T, Jha V, Neal B, Patrice HM, Okpechi I, et al. Worldwide access to treatment for end-stage kidney disease: a systematic review. Lancet. 2015 May 16;385(9981):1975–82.DOI: 10.1016/S0140-6736(14)61601-9. URL: https://pubmed.ncbi.nlm.nih.gov/25777665/

3. Lv JC, Zhang LX. Prevalence and disease burden of chronic kidney disease. In: Liu BC, Lan HY, Lv LL, editors. Renal Fibrosis: Mechanisms and Therapies. Singapore: Springer; 2019. p. 3–15. DOI:10.1007/978-981-13-8871-2_1URL: https://doi.org/10.1007/978-981-13-8871-2_1

4. Hiremath S. NephJC. 2024 [Internet]. The worldwide shadow of kidney disease. URL: http://www.nephjc.com/news/global-kidney-atlas

5. Global Burden of Disease Collaborative Network. Global Burden of Disease Study 2021(GBD 2021) [Internet]. Seattle, United States: Institute for Health Metrics and Evaluation (IHME) 2025. URL: https://www.healthdata.org/research-analysis/gbd

6. Sharma I, Bhattarai M, Sigdel MR. Global dialysis perspective: Nepal. Kidney360. 2022 Apr 27;3(7):1269–74. DOI: 10.34067/KID.0001672022. URL: https://pmc.ncbi.nlm.nih.gov/articles/PMC9337906/

7. Sharma D, Sharma T, Tang J. Nephrology in Nepal: How it started and where we are. Kidney360. 2025 Feb 25.DOI:10.34067/KID.0000000991 URL: https://pubmed.ncbi.nlm.nih.gov/40900674/

8. Shrestha S, Upadhyay HP, Shrestha P. Treatment adherence among patients undergoing hemodialysis in hospitals providing dialysis service of Bharatpur Nepal. Journal of Chitwan Medical College. 2023 Jun 30;13(2):36-40. URL: http://jcmc.com.np/jcmc/index.php/jcmc/article/view/1305

9. Dsouza B, Prabhu R, Unnikrishnan B, Ballal S, Mundkur SC, Chandra Sekaran V, et al. Effect of educational intervention on knowledge and level of adherence among hemodialysis patients: a randomized controlled trial. Global Health, Epidemiology and Genomics. 2023;2023(1):4295613. DOI: https://doi.org/ 10.1155/2023/4295613 .URL: https://onlinelibrary.wiley.com/doi/abs/10.1155/2023/4295613

10. LNU A, Rathee G, Sharma R, Dahiya S, Vaibhav F, Kumar P. Treatment adherence and factors influencing it in end-stage renal disease patients on maintenance hemodialysis: a study from a tertiary care hospital in North India. Cureus. 16(11):e73335. DOI: https://doi.org/10.7759/cureus.73335.URL: https://pmc.ncbi.nlm.nih.gov/articles/PMC11627191/

11. Feijão GMM, de Freitas Melo C. Treatment adherence of patients with chronic kidney disease: an integrative literature review. Trends in Psychol. 2020 Sept 1;28(3):399–418. DOI: https:10.1007/s43076-020-00031-5 URL: https://link.springer.com/article/10.1007/s43076-020-00031-5

12. Denhaerynck K, Manhaeve D, Dobbels F, Garzoni D, Nolte C, De Geest S. Prevalence and consequences of nonadherence to hemodialysis regimens. Am J Crit Care. 2007 May 1;16(3):222–35. DOI: 10.1046/j.1523-1755.2003.00064.x. URL: https://doi.org/10.4037/ajcc2007.16.3.222

13. Saran R, Bragg-Gresham JL, Rayner HC, Goodkin DA, Keen ML, Van Dijk PC, et al. Nonadherence in hemodialysis: associations with mortality, hospitalization, and practice patterns in the DOPPS. Kidney Int. 2003 July;64(1):254–62. DOI: https://doi.org/ 10.1046/j.1523-1755.2003.00064.x.URL: https://pubmed.ncbi.nlm.nih.gov/12787417/

14. Laradhi AO, Shan Y, Mansoor Al Raimi A, Hussien NA, Ragab E, Getu MA, et al. The association of illness perception and related factors with treatment adherence among chronic hemodialysis patients with cardio-renal syndrome in Yemen. Front Cardiovasc Med. 2025 Apr 30;12:1432648. DOI: 10.3389/fcvm.2025.1432648, URL: https://pmc.ncbi.nlm.nih.gov/articles/PMC12075227/

15. Naalweh KSh, Barakat MA, Sweileh MW, Al-Jabi SW, Sweileh WM, Zyoud SH. Treatment adherence and perception in patients on maintenance hemodialysis: a cross – sectional study from Palestine. BMC Nephrol. 2017 May 30;18:178. URL: https://link.springer.com/article/10.1186/s12882-017-0598-2

16. Seyyedrasooli A, Parvan K, Rahmani A, Rahimi Z. Effect of illness perception promoting interventions on treatment adherence in hemodialysis patients: A randomized controlled trial. URL: <https://www.sid.ir/paper/151565/en>

17. Cochran WG. Cochran 1977 Sampling techniques. 3rd ed. Harvard University; URL: https://www.jstor.org/stable/2240496

18. Kim Y, Evangelista LS, Phillips LR, Pavlish C, Kopple JD. The End-Stage Renal Disease Adherence Questionnaire (ESRD-AQ): testing the psychometric properties in patients receiving in-center hemodialysis. Nephrol Nurs J. 2010;37(4):377–93. PMID: 20830945.URL: https://pubmed.ncbi.nlm.nih.gov/20830945/

19. Dhimal M, Bista B, Bhattarai S, Dixit LP, Hyder MKA, Agrawal N, Rani M, Jha AK. Report of Non-Communicable Disease Risk Factors: STEPS Survey Nepal 2019. Kathmandu,Nepal; 2020. URL: https://www.who.int/docs/default-source/nepal-documents/ncds/ncd-steps-survey-2019-compressed.pdf

20. Nepal Living Standard Survey IV 2022/23 [Internet]. [cited 2025 Mar 26]. URL: http://data.nsonepal.gov.np/dataset/poverty-status-2023/resource/e2d52301-1c25-498b-8732-4326c62a2372

21. Belhmer FS, Al Amad MA, Albitahi MH, Babattah FK. Adherence to Treatment Regimens Among End-Stage Renal Disease Patients: A Cross-Sectional Study. Int J Nephrol Renovasc Dis. 2025;18:143–50. URL: https://www.tandfonline.com/doi/full/10.2147/IJNRD.S511221

22. Hussain F, Ashraf S, Arshad M, Rehman MM ur, Khan FS, Ahmad MA, et al. Knowledge, Adherence, and Perception of Patients on Maintenance Hemodialysis to Treatment Regimens at a Tertiary Care Hospital in Pakistan. Cureus [Internet]. 2023 Dec 30 [cited 2025 Nov 14];15(12). URL: https//cureus.com/articles/195146-knowledge-adherence-and-perception-of-patients-on-maintenance-hemodialysis-to-treatment-regimens-at-a-tertiary-care-hospital-in-pakistan

23. James M, Roy A, Antony E, George S. Impact of patient counseling on treatment adherence behavior and quality of life in maintenance hemodialysis patients. Saudi J Kidney Dis Transpl. 2021;32(5):1382–7. URL: https://journals.lww.com/sjkd/fulltext/2021/32050/impact_of_patient_counseling_on_treatment.20.aspx

24. Rozi NRB, Bin Wan Ali WASR, Bin Draman CR, Pasi HB, Rathor MY. Knowledge and Perceptions on End-of-life Care among End-stage Renal Disease Patients on Hemodialysis. Saudi Journal of Kidney Diseases and Transplantation. 2022 Oct;33(5):664. URL: https://journals.lww.com/sjkd/fulltext/2022/33050/knowledge_and_perceptions_on_end_of_life_care.7.aspx

25. AlHejaili F, Hashmi MN, Alsuwaida A, Ankawi GA, ALMehaideb SA, Alsuwaida AA, et al. Burden of Chronic Hemodialysis on the Ability to Work: Time for Action. Avicenna J Med. 2024 June 21;14(2):110–4. URL: https://www.thieme-connect.com/products/ejournals/html/10.1055/s-0044-1786869

26. Bazrafshan FD, Darvizeh Z, Banijamali SS. The relationship between hemodialysis patients’ treatment adherence, procrastination, and difficulty in emotion regulation: A cross-sectional study in southeast Iran. Front Psychol [Internet]. 2023 Jan 16 [cited 2025 Nov 16];13. URL: https://www.frontiersin.org/journals/psychology/articles/10.3389/fpsyg.2022.1041912/full
