# Supplementary material for: Factors influencing perceived importance of haemodialysis adherence among end stage kidney failure patients in tertiary care centres in Nepal
Source: PLoS One. 2026 Jun 17;21(6):e0351702. doi: 10.1371/journal.pone.0351702 (PMC13274901; doi:10.1371/journal.pone.0351702)
Supplement: Peer review history

# Peer review: Decision Letter 0

**DOI:** 10.1371/journal.pone.0351702.r001

**Authors:** Nik Hisamuddin Nik Ab. Rahman

26 Mar 2026

PONE-D-26-07737

Factors influencing perceived importance of hemodialysis adherence among end stage renal disease patients in tertiary care centers in Nepal

PLOS One

Dear Dr. Shrestha,

Thank you for submitting your manuscript to PLOS ONE. After careful consideration, we feel that it has merit but does not fully meet PLOS ONE’s publication criteria as it currently stands. Therefore, we invite you to submit a revised version of the manuscript that addresses the points raised during the review process.

Please submit your revised manuscript by May 10 2026 11:59PM. If you will need more time than this to complete your revisions, please reply to this message or contact the journal office at plosone@plos.org. When you're ready to submit your revision, log on to https://www.editorialmanager.com/pone/ and select the 'Submissions Needing Revision' folder to locate your manuscript file.

Please include the following items when submitting your revised manuscript:

- A letter that responds to each point raised by the academic editor and reviewer(s). You should upload this letter as a separate file labeled 'Response to Reviewers'.
- A marked-up copy of your manuscript that highlights changes made to the original version. You should upload this as a separate file labeled 'Revised Manuscript with Track Changes'.
- An unmarked version of your revised paper without tracked changes. You should upload this as a separate file labeled 'Manuscript'.

If you would like to make changes to your financial disclosure, please include your updated statement in your cover letter. Guidelines for resubmitting your figure files are available below the reviewer comments at the end of this letter.

If applicable, we recommend that you deposit your laboratory protocols in protocols.io to enhance the reproducibility of your results. Protocols.io assigns your protocol its own identifier (DOI) so that it can be cited independently in the future. For instructions see: https://journals.plos.org/plosone/s/submission-guidelines#loc-laboratory-protocols. Additionally, PLOS ONE offers an option for publishing peer-reviewed Lab Protocol articles, which describe protocols hosted on protocols.io. Read more information on sharing protocols at https://plos.org/protocols?utm_medium=editorial-email&utm_source=authorletters&utm_campaign=protocols.

As the corresponding author, your ORCID iD is verified in the submission system and will appear in the published article. PLOS supports the use of ORCID, and we encourage all coauthors to register for an ORCID iD and use it as well. Please encourage your coauthors to verify their ORCID iD within the submission system before final acceptance, as unverified ORCID iDs will not appear in the published article. Only the individual author can complete the verification step; PLOS staff cannot verify ORCID iDs on behalf of authors.

We look forward to receiving your revised manuscript.

Kind regards,

Nik Hisamuddin Nik Ab. Rahman

Academic Editor

PLOS One

Journal Requirements:

When submitting your revision, we need you to address these additional requirements.

1. Please ensure that your manuscript meets PLOS ONE's style requirements, including those for file naming. The PLOS ONE style templates can be found at

https://journals.plos.org/plosone/s/file?id=wjVg/PLOSOne_formatting_sample_main_body.pdf and

https://journals.plos.org/plosone/s/file?id=ba62/PLOSOne_formatting_sample_title_authors_affiliations.pdf

2.  In this instance it seems there may be acceptable restrictions in place that prevent the public sharing of your minimal data. However, in line with our goal of ensuring long-term data availability to all interested researchers, PLOS’ Data Policy states that authors cannot be the sole named individuals responsible for ensuring data access (http://journals.plos.org/plosone/s/data-availability#loc-acceptable-data-sharing-methods).

Data requests to a non-author institutional point of contact, such as a data access or ethics committee, helps guarantee long term stability and availability of data. Providing interested researchers with a durable point of contact ensures data will be accessible even if an author changes email addresses, institutions, or becomes unavailable to answer requests.

Before we proceed with your manuscript, please also provide non-author contact information (phone/email/hyperlink) for a data access committee, ethics committee, or other institutional body to which data requests may be sent. If no institutional body is available to respond to requests for your minimal data, please consider if there any institutional representatives who did not collaborate in the study, and are not listed as authors on the manuscript, who would be able to hold the data and respond to external requests for data access? If so, please provide their contact information (i.e., email address). Please also provide details on how you will ensure persistent or long-term data storage and availability.

3. If the reviewer comments include a recommendation to cite specific previously published works, please review and evaluate these publications to determine whether they are relevant and should be cited. There is no requirement to cite these works unless the editor has indicated otherwise.

[Note: HTML markup is below. Please do not edit.]

Reviewers' comments:

Reviewer's Responses to Questions

**Comments to the Author**

1. Is the manuscript technically sound, and do the data support the conclusions?

The manuscript must describe a technically sound piece of scientific research with data that supports the conclusions. Experiments must have been conducted rigorously, with appropriate controls, replication, and sample sizes. The conclusions must be drawn appropriately based on the data presented.

Reviewer #1: Yes

Reviewer #2: Partly

**********

2. Has the statistical analysis been performed appropriately and rigorously?

Reviewer #1: Yes

Reviewer #2: No

**********

3. Have the authors made all data underlying the findings in their manuscript fully available?

The PLOS Data policy requires authors to make all data underlying the findings described in their manuscript fully available without restriction, with rare exception (please refer to the Data Availability Statement in the manuscript PDF file). The data should be provided as part of the manuscript or its supporting information, or deposited to a public repository. For example, in addition to summary statistics, the data points behind means, medians and variance measures should be available. If there are restrictions on publicly sharing data—e.g. participant privacy or use of data from a third party—those must be specified.

Reviewer #1: Yes

Reviewer #2: No

**********

4. Is the manuscript presented in an intelligible fashion and written in standard English?

PLOS ONE does not copyedit accepted manuscripts, so the language in submitted articles must be clear, correct, and unambiguous. Any typographical or grammatical errors should be corrected at revision, so please note any specific errors here.

Reviewer #1: Yes

Reviewer #2: Yes

**********

5. Review Comments to the Author

Please use the space provided to explain your answers to the questions above. You may also include additional comments for the author, including concerns about dual publication, research ethics, or publication ethics. (Please upload your review as an attachment if it exceeds 20,000 characters)

Reviewer #1: This is a very practical and insightful study. Understanding what drives hemodialysis adherence in Nepal is incredibly important for improving patient care in tertiary centers.

I appreciate the straightforward methodology and the use of a validated tool like the ESRD-AQ. Your findings regarding the positive influence of family support (specifically, being accompanied by parents) are clear and offer valuable guidance for clinical practice.

I have no changes to request. Congratulations on completing a highly relevant and well-executed study.

Reviewer #2: This manuscript examines factors influencing patients’ perceptions regarding the importance of adherence to hemodialysis in two tertiary care centers in Nepal. The topic is relevant given the increasing burden of chronic kidney disease and the challenges of maintaining adherence to dialysis therapy in resource-limited settings.

However, the manuscript requires substantial revision to improve clarity, methodological transparency, and interpretation of results.

Major Comments

1. Conceptual clarity of the primary outcome

The central outcome variable is “perceived importance of adherence to dialysis.”

This raises several conceptual concerns:

• Perception does not necessarily translate into actual adherence behaviour

• The manuscript does not demonstrate why perceived importance alone is a meaningful outcome

• The ESRD-AQ instrument was originally designed to assess actual adherence behaviours, not only perceptions

The authors should:

• Clearly justify the conceptual framework

• Explain why perception is important independent of behaviour

• Discuss whether perception predicts attendance at dialysis sessions, medication adherence, or fluid restriction adherence

Without this clarification, the clinical significance of the study remains unclear.

2. Description and validation of the ESRD-AQ instrument

The manuscript lacks sufficient detail regarding the questionnaire.

The authors should clarify:

• Which domains of the ESRD-AQ were used

• Whether the questionnaire was translated into Nepali

• Whether cultural adaptation or validation was performed

• Whether reliability testing was conducted (e.g., Cronbach’s alpha)

Failure to address these issues raises concerns about the validity of the measurement.

3. Statistical analysis requires greater transparency

The statistical methods section is insufficiently detailed.

Please clarify:

• The specific regression model used to estimate adjusted prevalence ratios

• whether Poisson regression with robust variance or log-binomial regression was applied

• How variables were selected for inclusion in the multivariable model

• Whether multicollinearity was assessed

• Whether model fit was evaluated

These methodological details are necessary to ensure reproducibility and validity of the analysis.

4. Cross-sectional design and causal interpretation

Several statements in the Results and Discussion sections imply causal relationships.

Because this is a cross-sectional study, only associations can be inferred.

The authors should revise the text accordingly.

5. Strengthening the discussion

The discussion currently lacks sufficient engagement with existing literature.

The authors should compare their findings with studies from:

• India

• Pakistan

• Southeast Asia

• sub-Saharan Africa

where dialysis adherence barriers have been studied extensively.

Additionally, the discussion should address structural barriers, such as:

• travel distance to dialysis centres

• financial constraints

• dialysis availability

• healthcare workforce shortages

6. Language and editorial quality

The manuscript requires substantial language editing.

Numerous grammatical and typographical errors are present throughout the manuscript.

Professional English language editing is strongly recommended prior to resubmission.

See a Line-by-Line review below

Title

The title is acceptable but could be simplified.

Suggested alternative:

“Factors associated with perceived importance of haemodialysis adherence among patients with end-stage kidney disease (or chronic kidney failure) in Nepal.”

Abstract

Lines 18–22

The wording is unclear and contains multiple grammatical errors.

Example:

“Perception towards importance of adhering to hemodialysis affects treatment adherence.”

This statement requires citation and clarification.

Introduction

Lines 39–47

The introduction contains repetition and grammatical errors, including duplicated phrases.

Example:

“low-to middle-income patients’ needs renal replacement therapy…”

This sentence should be rewritten for clarity.

Line 48–49

“Renal transplantation relies highly on the intense availability…”

This statement is unclear and requires revision.

General comment

The introduction would benefit from:

• A clearer knowledge gap

• Explanation of why perception matters

Methods

Lines 68–70

“perception on of the importance of adherence”

This phrase appears multiple times and should be corrected to:

“perception of the importance of adherence.”

Study sites

The authors should explain why these two centres were selected and whether they are representative of dialysis services in Nepal.

Questionnaire

More detailed information is needed regarding the following aspects:

- Translation procedures

- Interviewer training

- Questionnaire administration

- Validation steps

Statistical Methods

The description of statistical analysis is insufficient. Please include the following information:

- Type of regression model used

- Criteria for variable inclusion

- Approach to handling missing data

Results

The results section is generally understandable but requires improvement in the following areas:

- Clearer presentation of tables

- Consistent reporting of confidence intervals

- A more organized narrative structure

Discussion

The discussion section should be significantly expanded. Important components that are currently missing include:

- A comparison with international studies on dialysis adherence

- Interpretation in the context of health system limitations in Nepal

- Acknowledgment of the study's limitations

Limitations

The following limitations should be explicitly stated:

1. Cross-sectional design

2. Bias from self-reported questionnaires

3. Limited generalizability beyond tertiary centers

4. Measurement of perception and practice, rather than actual adherence

**********

6. PLOS authors have the option to publish the peer review history of their article (what does this mean?). If published, this will include your full peer review and any attached files.

If you choose “no”, your identity will remain anonymous but your review may still be made public.

**Do you want your identity to be public for this peer review?**  For information about this choice, including consent withdrawal, please see our Privacy Policy.

Reviewer #1: **Yes:**  Ganesh Praneeth Roy Avula

Reviewer #2: No

**********

[NOTE: If reviewer comments were submitted as an attachment file, they will be attached to this email and accessible via the submission site. Please log into your account, locate the manuscript record, and check for the action link "View Attachments". If this link does not appear, there are no attachment files.]

To ensure your figures meet our technical requirements, please review our figure guidelines: https://journals.plos.org/plosone/s/figures

You may also use PLOS’s free figure tool, NAAS, to help you prepare publication quality figures: https://journals.plos.org/plosone/s/figures#loc-tools-for-figure-preparation.

NAAS will assess whether your figures meet our technical requirements by comparing each figure against our figure specifications.

Attachment Submitted filename: Perceived importance of hemodialysis adherence .doc

# Author comment: Author response to Decision Letter 1

**DOI:** 10.1371/journal.pone.0351702.r002

23 May 2026

PONE-D-26-07737

Factors influencing perceived importance of haemodialysis adherence among end stage kidney failure patients in tertiary care centres in Nepal

PLOS One

May 3, 2026

The Academic Editor,

Plos One

Re: Factors influencing perceived importance of hemodialysis adherence among chronic kidney failure patients in tertiary care centers in Nepal

Dear Dr. Rahman,

Thank you for considering publishing our manuscript. We also thank you and the reviewers for your constructive comments, as they have tremendously improved the quality of the manuscript. We have taken note of all the reviewers’ comments and made corrections accordingly.

Please find below a point-by-point response to each comment raised.

Thank you for your time and consideration.

Sincerely,

Pramita Shrestha

Response to the Line-by-Line review below

Title

The title is acceptable but could be simplified.

Suggested alternative:

“Factors associated with perceived importance of haemodialysis adherence among patients with end-stage kidney disease (or chronic kidney failure) in Nepal.”

Response: As suggested we changed the title to “Factors associated with perceived importance of haemodialysis adherence among patients with chronic kidney failure in Nepal.”

Abstract

Lines 18–22

The wording is unclear and contains multiple grammatical errors.

Example:

“Perception towards importance of adhering to hemodialysis affects treatment adherence.”

This statement requires citation and clarification.

Response: Line number 19-22. It has been restated as: Perception of the importance of adhering to dialysis helps to determine how patients interpret and interact with the influencing attitude, thoughts and behaviors towards adhering to dialysis.

Introduction

Lines 39–47

The introduction contains repetition and grammatical errors, including duplicated phrases.

Example:

“low-to middle-income patients’ needs renal replacement therapy…”

This sentence should be rewritten for clarity.

Response: Line number 41-47. The sentence was rewritten as:

Chronic Kidney Disease (CKD) is a significant global public health concern that can lead to chronic kidney failure, requiring costly treatments such as dialysis or renal transplantation for survival. Chronic renal failure contributes to burden in health care system, productivity and economic growth (1). Worldwide, an estimated 4.9 to 9.7 million chronic renal failure patients need kidney replacement therapy, with majority residing in low-to-middle income countries. (2,3). In Nepal, CKD accounted for approximately two per cent of total deaths and one per cent of total DALYs, placing a significant financial burden on patients, the health care system and society.

Line 48–49

“Renal transplantation relies highly on the intense availability…”

This statement is unclear and requires revision.

Response : At line number 48-51. The statement has been revised to: Renal transplantation requires substantial resources such as donor availability and long-term financial support. Therefore, dialysis is used as primary treatment option for patients with chronic renal failure.

General comment

The introduction would benefit from:

• A clearer knowledge gap

• Explanation of why perception matters

Response : Line number 63-72. Despite growing evidence on treatment adherence among hemodialysis patients, most studies have focused on clinical and socio-demographic factors, with limited exploration of patients’ perceptions regarding the importance of adherence, particularly in low- and middle-income countries like Nepal. Patients’ perceptions play a important role in shaping their adherence behavior, as they influence attitudes, motivation, and engagement with treatment. Understanding these perceptions is essential for developing targeted interventions to improve adherence outcomes. Therefore, this study aims to assess the factors influencing the perceived importance of adherence to hemodialysis among patients with chronic renal failure. The findings contribute evidence on patient’s perception regarding the importance of dialysis and may inform policies and health systems to plan and implement effective interventions that improve adherence.

Methods

Lines 68–70

“perception on of the importance of adherence”

This phrase appears multiple times and should be corrected to:

“perception of the importance of adherence.”

Response : The phrase was corrected to: “perception of the importance of adherence.”.

Study sites

The authors should explain why these two centers were selected and whether they are representative of dialysis services in Nepal.

Response: At line number 86-88: These tertiary hospitals were selected because they provide specialized care for patients with chronic renal failure undergoing hemodialysis and have patient flow from diverse geographical and socioeconomic backgrounds that enhance representativeness.

Questionnaire

More detailed information is needed regarding the following aspects:

- Translation procedures

- Interviewer training

- Questionnaire administration

- Validation steps

Response:

Line number 150-153: Translation procedures: The standard End stage Renal Disease Adherence Questionnaire was available in English language and translated into Nepali to ensure linguistic appropriateness. Translation was done by expert. The translated Nepali version was then retranslated into English by another translator not involved in initial translation. Differences observed were reviewed and solved by research team.

Line number 155-158: Interviewer training: The training for data collection was provided to the enumerators before starting data collection. Training was given on study objectives, inclusion and exclusion criteria of the patients, informed consent process, confidentiality and interview techniques to ensure consistency and minimize interviewer bias.

Questionnaire administration: Participants were selected based on predefined inclusion criteria. The questionnaire was administered in Nepali language. Data was collected using face to face interview that lasted for about 30- 35 minutes. Verbal and written consent was obtained from each of the participants prior to data collection and they were informed about the study objectives. Participants were informed about their rights to participate in study or not. Participant privacy was maintained throughout the study.

Validation steps:

Line number 160-163: Pretesting of the quantitative questionnaire was conducted among 17 hemodialysis patients of Methinkot Hospital, Kavrepalanchok, and patients from the surgery outpatient department of DHKUH visited for their fistula treatment. After pretesting, the tools were modified and the questions were restructured to simplify and make the participants understand and use them correctly. Content validity was ensured through expert review by nephrologists.

Statistical Methods

The description of statistical analysis is insufficient. Please include the following information:

- Type of regression model used

- Criteria for variable inclusion

- Approach to handling missing data

Response: Line number 169-182. Poisson regression was used to assess the association between perception on the importance of adherence to dialysis and sociodemographic, dialysis-related factors and accessibility related factors, adjusting for sex, residence, marital status, religion, educational status, occupation and family source of income. Prior to multivariate analysis, multicollinearity was assessed using the variation inflation factor. High multicollinearity with VIF>10 was seen in the variable frequency of dialysis sessions per week. Pairwise correlation was examined but did not show correlation of variable frequency of dialysis session per week with other exposure variables and therefore predicted this was due to combination of other independent variables. Therefore, frequency of dialysis session per week was excluded in final multivariate model .Crude and adjusted prevalence ratios were reported within 95% confidence interval, and statistical significance was decided if the p-value was <0.05. Data completeness was checked at the time of collection to minimize missing information so we did not have any missing data.

Results

The results section is generally understandable but requires improvement in the following areas:

- Clearer presentation of tables

- Consistent reporting of confidence intervals

- A more organized narrative structure

Response: The result section has been streamlined.

Discussion

The discussion section should be significantly expanded. Important components that are currently missing include:

- A comparison with international studies on dialysis adherence

- Interpretation in the context of health system limitations in Nepal

- Acknowledgment of the study's limitations

Response: Line number 255-263: This study found no significant association between perception of the importance of adherence to dialysis and factors including gender, residence, religion, education, current dialysis access site, comorbid condition, complications during dialysis, number of medications taken as prescribed, and intradialytic weight gain. A systematic review revealed that illness perception was associated with domains of adherence especially to diet and fluid restriction but not to dialysis sessions (30). A study from Palestine and Rawanda revealed age, gender and residence were positively associated with dialysis adherence score (15, 31). Even though, the Nepal Government has been offering free dialysis services since 2016, not all hemodialysis patients consistently follow their treatment plans. More than one fourth of the people have faced catastrophic health expenditure for dialysis (27,28).Studies suggest that a large proportion of chronic renal failure patients struggle with adherence to treatment regimen due to financial constraints, lack of awareness, and limited access to treatment facilities (29).The lack of association between these variables in the study could be due to a relatively small sample size and other possible residual confounders. We had done acknowledgement of study limitations 258-265.

Limitations

The following limitations should be explicitly stated:

1. Cross-sectional design

2. Bias from self-reported questionnaires

3. Limited generalizability beyond tertiary centers

4. Measurement of perception and practice, rather than actual adherence

Response: The given limitations were stated in line number 274-286. The study was conducted in only two tertiary care centres in Nepal, which may not have adequately represented all the hospitals throughout Nepal, which could affect the generalizability of results. Secondly, the cross-sectional design might have been limited in generating causal inference, as we were not able to know if the perception towards dialysis adherence was already improved, or it was improved only after getting exposed to the dialysis treatment-related exposures. The majority of the questions were self-reported by the participants, establishing social desirability or recall bias as a fourth limitation of the study. Also, the study might have residual confounders that might have distorted the effect measures. Moreover, the study primarily measured perception of patients on adherence and self-reported practices related to adherence, which may not completely reflect their actual treatment adherence behavior. This can be applied to qualitative studies; future studies may consider doing so for in-depth information on perceptions.

Attachment Submitted filename: Response to Reviewer_PONE-D-26-07737.pdf

# Editor report: Decision Letter 1

**DOI:** 10.1371/journal.pone.0351702.r003

**Authors:** Nik Hisamuddin Nik Ab. Rahman

31 May 2026

Factors influencing perceived importance of haemodialysis adherence among end stage kidney failure patients in tertiary care centres in Nepal

PONE-D-26-07737R1

Dear Dr. Shrestha,

We’re pleased to inform you that your manuscript has been judged scientifically suitable for publication and will be formally accepted for publication once it meets all outstanding technical requirements.

Within one week, you’ll receive an e-mail detailing the required amendments. When these have been addressed, you’ll receive a formal acceptance letter and your manuscript will be scheduled for publication.

An invoice will be generated when your article is formally accepted. Please note, if your institution has a publishing partnership with PLOS and your article meets the relevant criteria, all or part of your publication costs will be covered. Please make sure your user information is up-to-date by logging into Editorial Manager at Editorial Manager® and clicking the ‘Update My Information' link at the top of the page. For questions related to billing, please contact billing support.

If your institution or institutions have a press office, please notify them about your upcoming paper to help maximize its impact. If they’ll be preparing press materials, please inform our press team as soon as possible -- no later than 48 hours after receiving the formal acceptance. Your manuscript will remain under strict press embargo until 2 pm Eastern Time on the date of publication. For more information, please contact onepress@plos.org.

Kind regards,

Nik Hisamuddin Nik Ab. Rahman

Academic Editor

PLOS One

Additional Editor Comments (optional):

Reviewers' comments:

# Editor report: Acceptance letter

**DOI:** 10.1371/journal.pone.0351702.r004

**Authors:** Nik Hisamuddin Nik Ab. Rahman

PONE-D-26-07737R1

PLOS One

Dear Dr. Shrestha,

I'm pleased to inform you that your manuscript has been deemed suitable for publication in PLOS One. Congratulations! Your manuscript is now being handed over to our production team.

At this stage, our production department will prepare your paper for publication. This includes ensuring the following:

* All references, tables, and figures are properly cited

* All relevant supporting information is included in the manuscript submission,

* There are no issues that prevent the paper from being properly typeset

You will receive further instructions from the production team, including instructions on how to review your proof when it is ready. Please keep in mind that we are working through a large volume of accepted articles, so please give us a few days to review your paper and let you know the next and final steps.

Lastly, if your institution or institutions have a press office, please let them know about your upcoming paper now to help maximize its impact. If they'll be preparing press materials, please inform our press team within the next 48 hours. Your manuscript will remain under strict press embargo until 2 pm Eastern Time on the date of publication. For more information, please contact onepress@plos.org.

You will receive an invoice from PLOS for your publication fee after your manuscript has reached the completed accept phase. If you receive an email requesting payment before acceptance or for any other service, this may be a phishing scheme. Learn how to identify phishing emails and protect your accounts at https://explore.plos.org/phishing.

If we can help with anything else, please email us at customercare@plos.org.

Thank you for submitting your work to PLOS ONE and supporting open access.

Kind regards,

PLOS ONE Editorial Office Staff

on behalf of

Professor Dr Nik Hisamuddin Nik Ab. Rahman

Academic Editor

PLOS One
